# Janus metagrating for tailoring direction-dependent wavefronts

**DOI:** 10.1515/nanoph-2025-0140

**Published:** 2025-05-22

**Authors:** Zhen Tan, Jianjia Yi, Shah Nawaz Burokur

**Affiliations:** School of Information Science and Technology, Nantong University, Nantong, 226019, China; School of Information and Communications Engineering, Xi’an Jiaotong University, Xi’an, 710049, China; LEME, Univ Paris Nanterre, Ville d’Avray, 92410, France

**Keywords:** metagratings, sparse metasurfaces, asymmetric absorption and reflection

## Abstract

Janus metasurfaces have emerged as a promising platform to enable independent wave manipulation by fully exploiting the inherent propagation direction of electromagnetic waves. These structures allow achieving distinct wavefront functionalities based on the direction of wave propagation. Concurrently, metagratings have gathered significant attention as an innovative design scheme for wavefront manipulation, particularly in addressing the low efficiency issue commonly associated with conventional metasurfaces. This study introduces Janus metagratings as a means for tailoring efficient, direction-dependent absorption and reflection. Utilizing established analytical models, a precise analysis of diffraction modes is conducted in transmissive metagratings, facilitating asymmetric wavefront manipulation under the two incidence directions. By arranging distinct meta-atoms with specific load impedances on the upper and lower layers of the metagrating, efficient asymmetric wave responses are achieved. The design methodology is validated through full-wave simulations, which demonstrate strong consistency with theoretical predictions. Additionally, a Janus metagrating prototype is fabricated and tested in the microwave frequency regime, validating the direction-dependent wavefronts tailoring characteristics. The proposed design methodology offers a versatile platform for asymmetric propagation and advanced systems in future wireless and optical communication applications.

## 1 Introduction

Metasurfaces, a category of two-dimensional structured metamaterials comprising subwavelength meta-atoms organized in spatially varying patterns, offer a remarkable degree of flexibility in manipulating electromagnetic wavefronts, surpassing the capabilities of natural materials [1], [2], [3], [4]. The attention directed toward metasurfaces stems from their potential to revolutionize diverse applications in electromagnetism and optics, achieved through tailored control of amplitude [5], phase [6], and polarization [7]. Consequently, a broad spectrum of applications has been demonstrated, including anomalous reflection [8], anomalous refraction [9], beam splitting [10] and absorption [11]. Within this landscape, Janus metasurfaces have emerged as a particularly promising avenue for precise control and versatility in manipulating electromagnetic waves [12], [13]. Named after the Roman deity Janus, who is depicted with two faces gazing in opposite directions, Janus metasurfaces exhibit asymmetric functionalities upon the illumination direction that give rise to unique optical properties [14], [15], [16], [17].

In parallel, achieving high efficiency in metasurface wavefront manipulation has been a longstanding challenge [18]. Traditional phase-gradient metasurfaces, devices based on the generalized Snell’s law, encounter low efficiencies due to a mismatch between the wave impedance of outgoing and incident waves [19], which becomes particularly problematic with significant disparities in propagation angles between outgoing and incident waves [20]. Huygens metasurfaces, offering independent control over electric and magnetic responses, offer a potential solution to the wave impedance mismatch issue [21], [22]. However, their intricate multilayer structures incorporating metal vias often pose considerable implementation challenges [23]. Fortunately, inspiration drawn from the diffraction control of gratings has led to the design concept of metagratings, enabling wavefront manipulation with nearly 100 % efficiency through a sparse and straightforward design [24], [25], [26]. As such, metagratings [27], [28], [29], [30], [31], [32], [33], [34], [35], [36] hold broad potentials for next-generation wave manipulation applications owing to their low-profile attributes compared to three-dimensional metagrating structures [37], [38], [39].

Assymetric functionalities can attract great interests due to the high degree of integration and wavefronts tailoring possibilities. Assymetric diffraction has been achieved in the terahertz regime using an all-dielectric additively manufactured metagrating [40]. In this work, a bi-functional metagrating exhibiting anomalous refraction and beam splitting with efficiencies exceeding 80 % was explained using simplified modal methods. To date, in the microwave domain, studies have primarily concentrated on a single illumination direction and asymmetric functionalities have not been investigated in metagratings in comparison to dense metasurfaces. Addressing bidirectional illuminations will lead to potentials in integrating multiple functionalities in metagratings, thus enabling a single device to integrate more diverse wavefront manipulation capabilities for modern communication systems. Here, we introduce and validate the concept of wavefront engineering based on the propagation direction using Janus metagratings comprised of bilayered meta-atoms sparsely arranged on a dielectric substrate. It is demonstrated that by strategically designing a meta-atom on each side of the supercell (periodic unit cell), the reflection diffraction order and transmission diffraction order can be efficiently controlled in response to illumination directions. As a proof-of-concept demonstration, a device with high-efficiency absorption feature under forward illumination and reflection under backward illumination is experimentally validated in the microwave frequency range to substantiate the design principle and the practicality of the method. Our proposed methodology highlights a fundamentally new approach to direction-dependent manipulation of electromagnetic wavefronts, enabling asymmetric far-field wave manipulation and enhancing the versatility in tailoring wavefronts.

## 2 Janus metagrating for efficient asymmetric absorption and reflection

### 2.1 Theory

**Figure 1:**
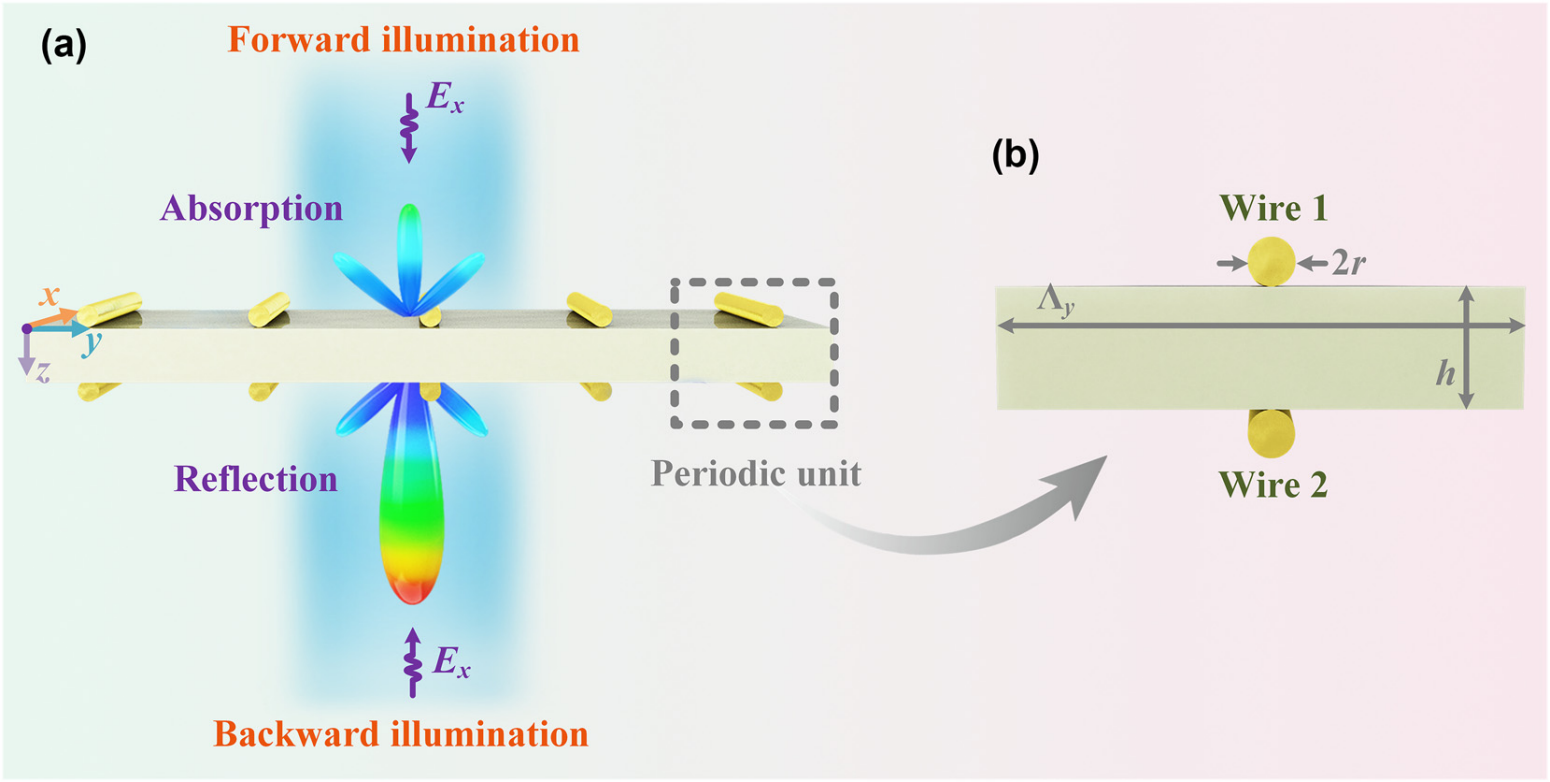
Schematic illustration of the Janus metagrating. (a) Direction-dependent electromagnetic wavefronts manipulation. (b) Side view of the supercell (periodic unit).

The schematic representation of the proposed Janus metagrating, designed to tailor high-efficiency asymmetric absorption and reflection, is depicted in Figure 1(a). When illuminated normally by a TE-polarized electromagnetic wave, the strategic arrangement of wires with specific load impedance densities on the upper and lower faces of the metagrating enables to achieve absorption under forward illumination and reflection under backward illumination. The considered metagrating is a two-dimensional structure that remains invariant along the *x*-axis and exhibits periodic alignment along the *y*-axis. Figure 1(b) depicts the side view of the supercell of period Λ_*y*_ composing the metagrating, where two conducting wires with radius *r* are positioned on each face of the dielectric substrate with thickness *h*, denoted by their coordinates as (*y*_1_, *z*_1_) = (0, −*h*) and (*y*_2_, *z*_2_) = (0, 0).

Given that both air and the dielectric substrate are non-magnetic media, the wave numbers and wave impedances in these media are respectively expressed as $k_{0}=\omega \sqrt{\mu_{0}\varepsilon_{0}}$ , $\eta_{0}=\sqrt{\mu_{0}/\varepsilon_{0}}$ , $k_{s}=\omega \sqrt{\mu_{0}\varepsilon_{s}}$ , and $\eta_{s}=\sqrt{\mu_{0}/\varepsilon_{s}}$ . *μ*_0_ and *ε*_0_ denote the permeability and permittivity of vacuum, respectively, and *ε*_*s*_ = *ε*_*r*_ *ε*_0_ is the permittivity of the dielectric substrate, with *ε*_*r*_ being the relative permittivity. For the diffraction modes within the metagrating, *ξ*_*m*_ = 2*πm*/Λ_*y*_ and $\beta_{m}=\sqrt{k_{0}^{2}-\xi_{m}^{2}}$ represent the tangential and normal components of the wavevector of the plane waves, respectively. Let $E_{m}^{\mathrm{UF}}$ and $E_{m}^{\mathrm{LF}}$ denote the amplitudes of the *m*th order diffraction mode in the upper and lower spaces, respectively, under forward illumination of the metagrating. Similarly, $E_{m}^{\mathrm{UB}}$ and $E_{m}^{\mathrm{LB}}$ represent the amplitudes of the *m*th order diffraction mode in the upper and lower spaces, respectively, under backward illumination. The comprehensive analytical expressions for $E_{m}^{\mathrm{UF}}$ , $E_{m}^{\mathrm{LF}}$ , $E_{m}^{\mathrm{UB}}$ and $E_{m}^{\mathrm{LB}}$ are developed in Note S1, Supporting Information. For simplicity, we select the period Λ_*y*_ of the metagrating such that Λ_*y*_ < *λ*_0_, ensuring that only the 0th diffraction mode propagates, while other diffraction modes are evanescent. The amplitudes of these 0th diffraction modes in the upper and lower spaces of the metagrating under forward and backward illuminations can be represented as $E_{0}^{\mathrm{UF}}=E_{in}A^{\mathrm{UF}}$ , $E_{0}^{\mathrm{LF}}=E_{in}A^{\mathrm{LF}}$ , $E_{0}^{\mathrm{UB}}=E_{in}A^{\mathrm{UB}}$ and $E_{0}^{\mathrm{LB}}=E_{in}A^{\mathrm{LB}}$ , where *E*_in_ is the amplitude of the incident field. Consequently, the Janus metagrating can be configured with distinct functionalities by assigning different values to *A* ^UF^, *A* ^LF^, *A* ^UB^ and *A* ^LB^. Specifically, when tailoring absorption under forward illumination and reflection under backward illumination, the assignments are $A^{\mathrm{UF}}=0,A^{\mathrm{LF}}=0,A^{\mathrm{UB}}=0\;\text{and}\;A^{\mathrm{LB}}=e^{j\varphi_{\mathrm{LB}}}$ , where $\varphi_{\mathrm{LB}}\in \left[0,2\pi \right)$ . Subsequently, the following relationship for calculating the required parameters of the Janus metagratings can be obtained as (for the detailed derivation, refer to Note S1, Supporting Information)

(1) $$\frac{\eta_{0}}{\Lambda_{y}}\left(\frac{1+R_{0}}{p^{\prime }}-\frac{T_{0}}{p^{\prime \prime }}\right)-\left(\frac{q^{\prime }}{p^{\prime }}-\frac{q^{\prime \prime }}{p^{\prime \prime }}\right)Z_{\mathrm{mutual}}=0$$

where $p^{\prime }=2\left[\left(1+R_{0}\right)R_{0}-T_{0}^{2}\right]/\left[{\left(1+R_{0}\right)}^{2}-T_{0}^{2}\right]$ , $q^{\prime }=2T_{0}/\left[{\left(1+R_{0}\right)}^{2}-T_{0}^{2}\right]$ , $p^{\prime \prime }=2T_{0}\left(1+e^{j\varphi_{\mathrm{LB}}}\right)/\left[{\left(1+R_{0}\right)}^{2},-T_{0}^{2}\right]$ , $q^{\prime \prime }=2\left[\left(1+R_{0}\right)\left(R_{0}-e^{j\varphi_{\mathrm{LB}}}\right)-T_{0}^{2}\right]/\left[{\left(1+R_{0}\right)}^{2}-T_{0}^{2}\right]$ and

(2) $$Z_{\mathrm{mutual}}=\frac{k_{0}\eta_{0}}{2\Lambda_{y}}\overset{\infty }{\underset{m=-\infty }{\sum }}\frac{T_{m}}{\beta_{m}}$$

with $R_{m}=j\left(1-\rho_{m}^{2}\right)\sin\left(\beta_{sm}h\right)/\left[2\rho_{m}\cos\left(\beta_{sm}h\right)+j\left(1+\rho_{m}^{2}\right)\right.\left.\sin\left(\beta_{sm}h\right)\right]$ and $T_{m}=2\rho_{m}/\left[2\rho_{m}\cos\left(\beta_{sm}h\right)+j\left(1+\rho_{m}^{2}\right)\right.\left.\sin\left(\beta_{sm}h\right)\right]$ are the corresponding Fresnel’s reflection and transmission coefficients [32], where $\beta_{sm}=\sqrt{k_{s}^{2}-\xi_{m}^{2}}$ and *ρ*_*m*_ = *β*_*sm*_/*β*_*m*_ (where *m* denotes the *m*th diffraction order of the metagratings). To solve Equation (1), which contains the three unknown variables Λ_*y*_, *h* and *φ*_LB_, we define the left-hand side of Equation (1) as a complex quantity $J=\frac{\eta_{0}}{\Lambda_{y}}\left(\frac{1+R_{0}}{p^{\prime }}-\frac{T_{0}}{p^{\prime \prime }}\right)-\left(\frac{q^{\prime }}{p^{\prime }}-\frac{q^{\prime \prime }}{p^{\prime \prime }}\right)Z_{\mathrm{mutual}}$ . The solution of this equation is therefore reduced to simultaneously satisfying the real and imaginary components of *J*, which is $ℜ\left(J\right)=0$ and $ℑ\left(J\right)=0$ . This formulation yields to a system of two equations with three unknowns $\left(\Lambda_{y},h\;\text{and}\;\varphi_{\mathrm{LB}}\right)$ . Consequently, the value of one of the three parameters can be arbitrarily fixed such that the values of the remaining two can be calculated.

### 2.2 Design and simulation

First, let us consider the choice of determination for the metagrating supercell. Although there is no strict limitation on the choice of parameters according to Equation (1), certain inappropriate choices may render practical implementation complex or impossible, such as when the real part of the impedance density is negative. Therefore, we adopt the selection strategy for the parameters involves by considering parameters ensuring practical feasibility: the structure must maintain sparsity, *i.e.*, the period length Λ_*y*_ should not be less than *λ*_0_/2, and finally, for a practical implementation, the real part of the impedance density should not be negative. Following judicious parameter screening, the period of the metagrating is chosen as Λ_*y*_ = 16 mm. Subsequently, applying $ℜ\left(J\right)=0$ and $ℑ\left(J\right)=0$ , the thickness of the substrate *h* = 2.53 mm and the parameter *φ*_LB_ = 1.8504 rad can be respectively calculated, meeting the low-profile feature of metagratings (*h* is smaller than *λ*_0_/10). Then, the required load impedance densities of the two wires can be calculated using (more details can be found in Note S1, Supporting Information)

(3) $$Z_{\text{U}}=\frac{\eta_{0}}{\Lambda_{y}}\frac{1+R_{0}}{p^{\prime }}-Z_{\mathrm{self}}-\frac{q^{\prime }}{p^{\prime }}Z_{\mathrm{mutual}}$$

(4) $$Z_{L}=\frac{\eta_{0}}{\Lambda_{y}}\frac{T_{0}}{p^{\prime }}-Z_{\mathrm{self}}-\frac{p^{\prime }}{q^{\prime }}Z_{\mathrm{mutual}}$$

with

(5) $$\begin{array}{ll} Z_{\mathrm{self}} & =\frac{\eta_{0}\left(1+R_{0}\right)}{2\Lambda_{y}}\\ & \;+j\frac{\eta_{0}}{\lambda_{0}}\left\{\overset{\infty }{\underset{m=1}{\sum }}\left[\frac{2\pi \left(1+R_{m}\right)}{j\Lambda_{y}\beta_{m}}-\frac{1}{m}\right]-\ln\left(\frac{2\pi r}{\Lambda_{y}}\right)\right\} \end{array}$$

Since the substrate thickness *h* of the metagrating is determined based on a rigorous solution of the equations, it may not correspond to commercially available values. To consider a more realistic physical implementation, the calculated substrate thickness *h* = 2.53 mm is adjusted to a commercially available value of 2.5 mm. After fixing *h* to 2.5 mm, the number of unknowns becomes less than the number of equations. Thus, the least squares method is employed using the *lsqnonlin* solver in Matlab to find the optimal solution for the impedance densities *Z*_U_ and *Z*_L_ that simultaneously maximize both absorption under forward illumination and reflection under backward illumination. Finally, for *h* = 2.5 mm, the parameters are calculated as *φ*_LB_ = 2.3000 rad, *Z*_U_ = −*j*4.3000 *η*/*λ*, and *Z*_L_ = (0.3500 − *j*4.0000) *η*/*λ*, respectively.

**Figure 2:**
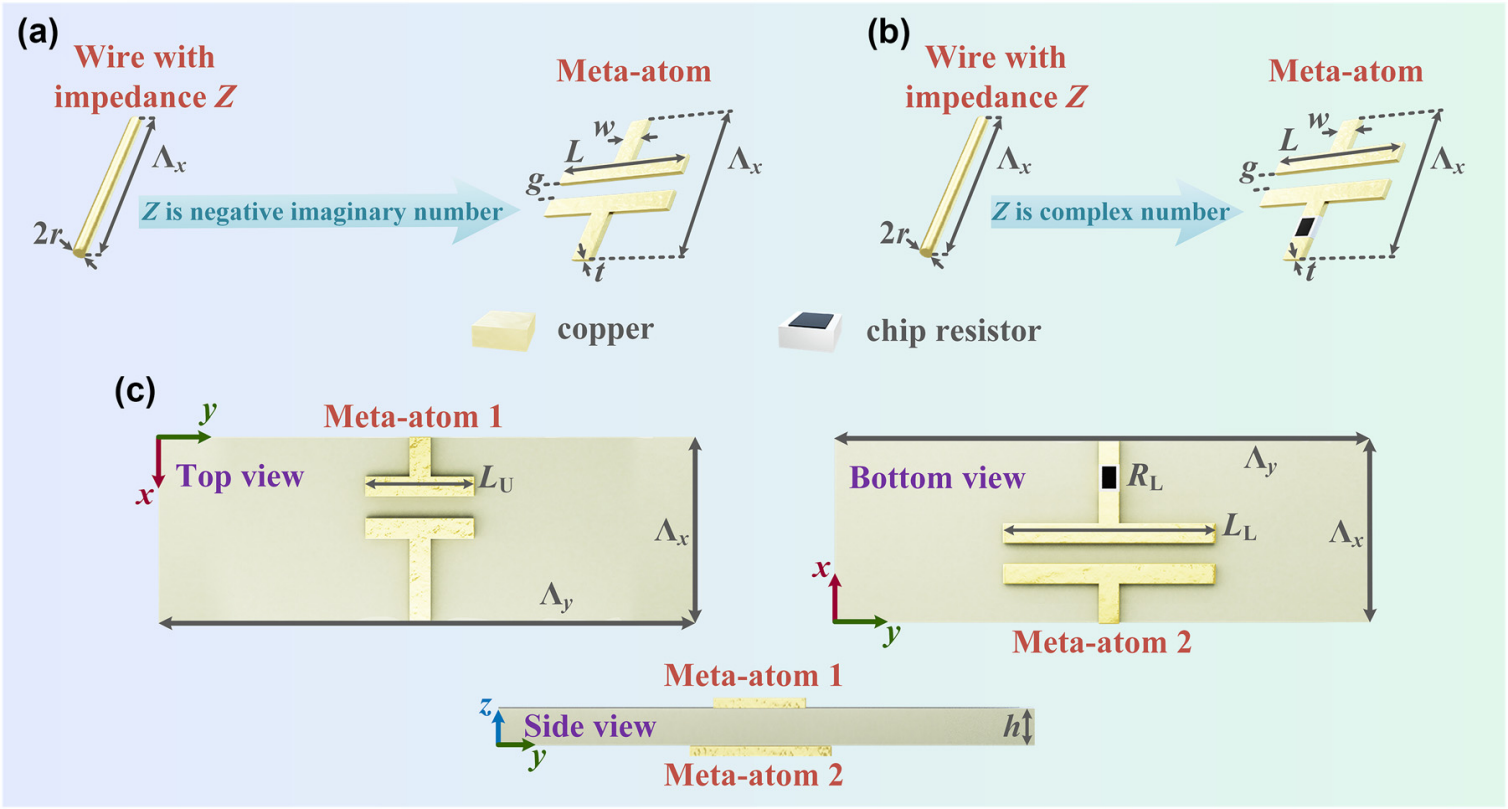
Composition of the Janus metagrating supercell. (a) Realization of a meta-atom with negative imaginary number load impedance density using a microstrip capacitor. (b) Realization of a meta-atom with complex number load impedance density using a microstrip capacitor in series with a chip resistor. (c) Individual views of the Janus metagrating supercell.

Next, we consider using meta-atoms with specific structures to replace the wires bearing the calculated load impedances. The meta-atom in the upper layer of the metagrating is constructed using a microstrip line capacitor to achieve the negative imaginary impedance density, as illustrated in Figure 2(a). When a cylindrical wire with a specific load impedance is transformed into a microstrip line capacitor structure, the width of the microstrip line is taken as *w* = 4*r*, with *r* being the radius of the cylindrical wire [41]. In order to take into account the accuracy of the PCB technology fabrication, the value of 0.2 mm is set to *w*. For the meta-atom on the upper layer requiring complex number impedance densities, a microstrip line capacitor in series with a 01005 series chip resistor (0.4 mm long and 0.2 mm wide) is utilized, as depicted in Figure 2(b). The individual views of the constructed supercell of the metagrating are shown in Figure 2(c).

In order to ensure the uniformity of the load impedance density, the period of the supercell along the *x*-axis takes the value of Λ_*x*_ = *λ*_0_/10 = 3 mm. Such configuration guarantees that the meta-atom arrangement in the *x*-direction maintains subwavelength characteristics throughout the operational bandwidth. Then, the length *L* of the microstrip capacitor can be found for the required imaginary part of the impedance density $ℑ\left(Z\right)$ as $L=\eta_{0}\lambda_{0}/\left\{4ℑ\left(Z\right)\varepsilon_{\mathrm{eff}}\Lambda_{x}\ln\left[\sin\left(\pi w/2\Lambda_{x}\right)\right]\right\}$ [33], where *ε*_eff_ = (*ε*_0_ + *ε*_*s*_)/2 is the effective permittivity at the interface between air and the substrate. The resistance value *R* can be obtained according to the real part of impedance density $ℜ\left(Z\right)$ as $R=ℜ\left(Z\right)\Lambda_{x}$ . Finally, the capacitor arm length and resistance of the corresponding meta-atoms for *h* = 2.5 mm are calculated as *L*_U_ = 3.86 mm, *L*_L_ = 4.15 mm and *R*_L_ = 13.19 Ω, as detailed in Table 1.

**Table 1:** Parameters of the Janus metagrating supercell.

Parameters	Calculated	Optimized/experimental
*h* (mm)	2.5	2.5
*L*_U_ (mm)	3.86	3.60
*L*_L_ (mm)	4.15	4.00
*R*_L_ (Ω)	13.19	11.50

**Figure 3:**
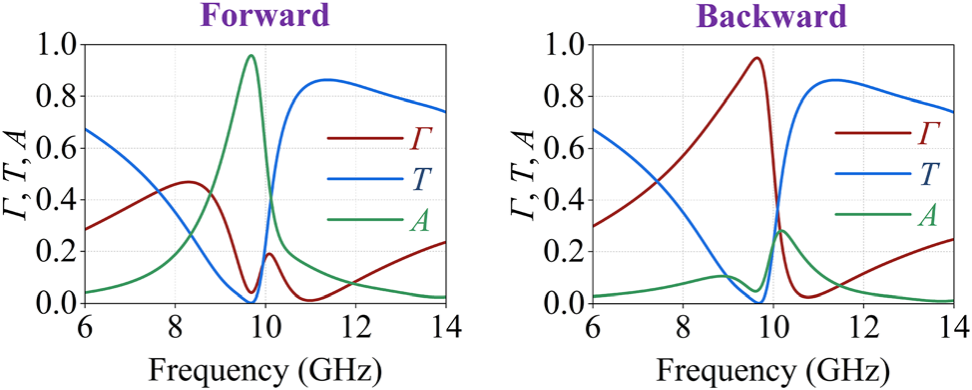
Simulation results of the Janus metagrating supercell with the calculated parameters: *h* = 2.5 mm, *L*_U_ = 3.86 mm, *L*_L_ = 4.15 mm and *R*_L_ = 13.19 Ω, demonstrating asymmetric absorption and reflection.

Next, a full-wave simulation of the metagrating supercell is conducted and periodic boundaries are used. The simulation results for both forward and backward illuminations of the Janus metagrating supercell for *h* = 2.5 mm are depicted in Figure 3, where *Γ* denotes reflection, *T* denotes transmission, and absorption is calculated using the relation *A* = 1 − *Γ* − *T*. The maximum absorption under forward illumination is 95.77 % at 9.68 GHz, and the maximum reflection under backward illumination is 94.91 % at 9.64 GHz. From the simulation results, we observe that the wavefront manipulation of the Janus metagrating deviates slightly from our design expectations in terms of operation frequency and efficiency. These deviations are mainly attributed to two factors: first, the copper-based microstrip capacitor structure inevitably introduces a small amount of losses (real part of the impedance density) and second, there is a mutual coupling between the upper and lower meta-atoms, due to their close proximity.

It should be noted that all the parameters of the metagrating so far have been obtained through a purely analytical procedure without the aid of any numerical optimization, and the results are reasonably close to expectations. A simple parameter sweep on the parameters *L* and *R* can complete the optimization through a full-wave electromagnetic solver, demonstrating the effectiveness and convenience of the proposed analytical framework. Additionally, the design can incorporate meta-atoms with asymmetric positions on the upper and lower layers of the substrate. This approach increases the distance between the upper and lower meta-atoms, reducing their coupling and resulting in simulation outcomes closer to the expected values. Moreover, this method enhances design flexibility by alleviating constraints imposed by substrate thickness, allowing for more adaptable substrate thickness choices, as detailed in Note S2, Supporting Information.

## 3 Experiment and discussion

For a proof-of-concept validation, the printed circuit board (PCB) technique is considered for fabrication, with resistance values approached using the commercial 01005 series chip resistor that have physical dimensions 0.2 mm × 0.4 mm. Considering the physical implementation of the device, the commercially available substrate thickness *h* = 2.5 mm is chosen, and the parameters *L* and *R* are then optimized numerically in HFSS to achieve optimal absorption under forward illumination and reflection under backward illumination at 10 GHz. Finally, the parameters used for the fabrication of the metagrating prototype are given in Table 1.

**Figure 4:**
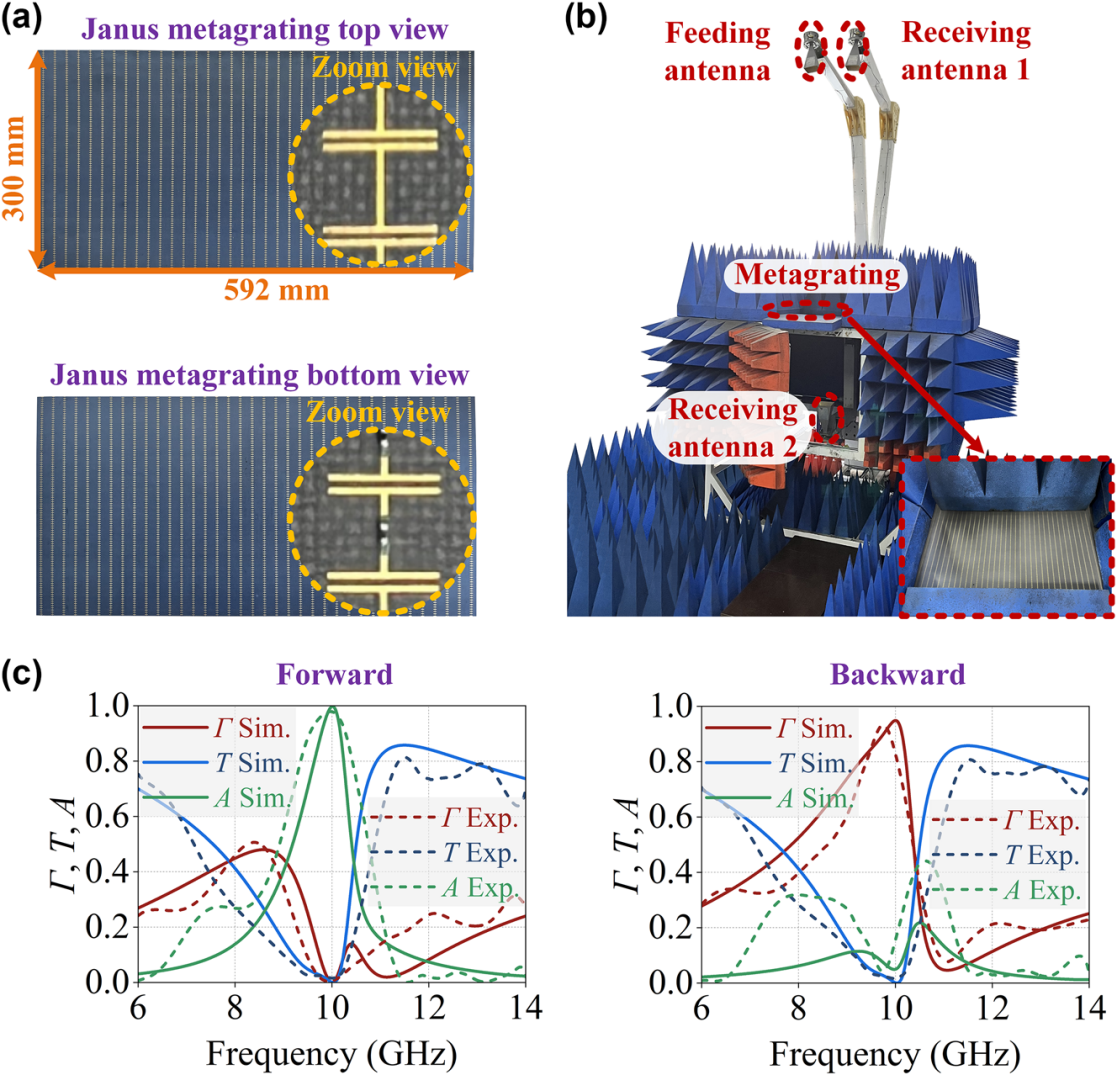
Experimental characterization of the Janus metagrating. (a) Top and bottom views of the fabricated Janus metagrating. (b) Set-up demonstrating the measurement of the reflection and transmission coefficients for the Janus metagrating. A standard horn antenna is used as a transmitter 2.5 m far away of the metagrating to create the incident quasi-plane wave. Two other standard horn antennas are used as receivers to measure the reflected and transmitted fields. (c) Simulated and measured reflection *Γ*, transmission *T*, and absorption *A* responses under forward and backward illuminations.

The fabricated Janus metagrating prototype, displayed in Figure 4(a), consists of 100 × 37 supercells in *x*- and *y*-directions, corresponding to dimensions of 300 mm × 592 mm. The photograph of the measurement setup is shown in Figure 4(b), where the sample under test is surrounded by microwave absorbing materials. A standard horn antenna is used to illuminate the sample at 2.5 m away and another two antennas are used as receivers either in the reflection or transmission semi-space. Due to practical limitations in order to avoid collision between two antennas, the reflection coefficient was measured for an incidence angle *θ*_*i*_ = −3° at a receiving angle *θ*_*r*_ = 3°. The simulation performed with the optimized parameters and measurement are compared in Figure 4(c), where a good overall agreement can be observed.

To further evaluate the performance of the Janus metagrating, the fractional absorption bandwidth and fractional reflection bandwidth for efficiency greater than 80 % are evaluated. The fractional absorption bandwidth is determined to be 6 % and 10.6 % in simulation and experiment, respectively, while the fractional reflection bandwidth is found to be 9.4 % and 6.3 % in simulation and experiment, respectively. To further assess the absorption bandwidth-to-thickness ratio performance of the Janus metagratings, we compare their performance against the theoretical limit established for transparent layers in Ref. [42]. Generally, achieving a broader absorption bandwidth necessitates the use of thicker substrates. The normalized absorption bandwidth-to-thickness ratio *η* for the Janus metagrating was calculated to be 0.054 (simulation) and 0.098 (experiment), as detailed in Note S3 (Supporting Information). These results indicate that, despite some unavoidable fabrication tolerances and limited size of the metagrating sample, the fabricated Janus metagrating demonstrates favorable wave absorption characteristics under forward incidence and qualitative reflection characteristics under backward illumination at the operating frequency of 10 GHz.

## 4 Conclusions

The presented low-profile Janus metagrating, using a sparse array of meta-atoms with a simple structure, offer efficient direction-dependent wavefront manipulation capabilities, achieving asymmetric absorption and reflection for forward and backward illuminations, respectively. A detailed design methodology is provided for the analytical analysis and for the calculation of the required load impedance density based on the desired direction-dependent wavefront manipulation function, leading to the elementary meta-atom construction. Simulated and experimental results validate the design methodology and the asymmetric responses, aligning well with theoretical predictions. Compared to previous scattering structures using traditional metasurfaces, Janus metagratings with an inter-element distance larger than half-wavelength demonstrate an intriguing design paradigm, suggesting the possibility of achieving current wavefront designs with more sparse and simple structures. The proposed design methodology further suggests promising utility for future designs of planar sparse metasurfaces with high-efficiency asymmetric performances to foster the development of direction-dependent manipulation and processing of electromagnetic waves.

The generality of the theoretical approach enables the design of Janus metagratings with advanced functionalities beyond previous attempts at asymmetric absorption and reflection. This paradigm can be expanded to achieve broader operation by considering more diffraction orders when increasing the number of meta-atoms in a supercell, offering more degrees of freedom in the design process. Additionally, the proposed concept can be scaled to higher frequencies due to the sparse arrangement of the meta-atoms, where ultralow-profile and planar multifunctional devices are desirable in wireless communication systems. Furthermore, the proposed Janus metagrating design offers significant flexibility in implementation. Specifically, lumped tunable capacitive elements can be directly integrated in the microstrip structures to allow for straightforward extension to reconfigurable Janus metagratings, thereby enabling dynamic direction-dependent wavefront control.

## Supplementary Material

Supplementary Material Details
