# Corrigendum to: Janus metagrating for tailoring direction-dependent wavefronts

**DOI:** 10.1515/nanoph-2025-0454

**Published:** 2025-09-22

**Authors:** Zhen Tan, Jianjia Yi, Shah Nawaz Burokur

**Affiliations:** School of Information Science and Technology, 66479 Nantong University, Nantong, 226019, China; School of Information and Communications Engineering, Xi’an Jiaotong University, Xi’an, 710049, China; LEME, Univ Paris Nanterre, Ville d’Avray, 92410, France

Following the publication of our paper [1], the authors wish to clarify two key parameters that were inadvertently omitted from both the main text and the Supplementary Material:

– The operational frequency of the designed metagrating is 10 GHz.
– The dielectric substrate material used in both simulations and experiments is F4BM300, with a relative permittivity of *ε*_*r*_ = 3 and a loss tangent of tan*δ* = 0.0007.

These details are essential for the reproduction of the reported results. We apologize for any inconvenience this omission may have caused.
