# Supplementary material for: Janus metagrating for tailoring direction-dependent wavefronts
Source: Nanophotonics. 2025 May 22;14(12):2229–36. doi: 10.1515/nanoph-2025-0140 (PMC12147549; doi:10.1515/nanoph-2025-0140)
Supplement: Supplementary file 1 — Supplementary Material Details [file j_nanoph-2025-0140_suppl_001.pdf]

Supplementary Material

Title: Janus Metagrating for Tailoring Direction-Dependent Wavefronts

Zhen Tan, Jianjia Yi* and Shah Nawaz Burokur*

*Corresponding authors: **Jianjia Yi**, School of Information and Communications Engineering, Xi'an Jiaotong University, Xi'an, 710049, China, e-mail: jianjia.yi@xjtu.edu.cn; **Shah Nawaz Burokur**, LEME, Univ Paris Nanterre, Ville d'Avray, 92410, France, e-mail: sburokur@parisnanterre.fr.
Zhen Tan, School of Information Science and Technology, Nantong University, Nantong, 226019, China, China, e-mail: zhzhtan@hotmail.com.

Text S1. Analytical design of the Janus metagrating

Here, let us consider a generalized configuration for the supercell where the wires are asymmetrically positioned on the upper and lower layers within the supercell. The two wires are horizontally spaced by a distance d, with coordinates $(y_1, z_1) = (0, -h)$ and $(y_2, z_2) = (d, 0)$, as illustrated in **Figure S1**. For the case of symmetric top and bottom positions design presented in the main text, the spacing is simplified to $d = 0$.

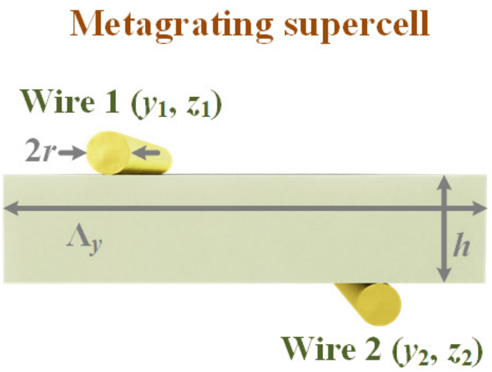

Figure S1. Janus metagrating supercell containing two wires with asymmetrically positioned upper and lower meta-atoms.

Under the condition of forward normal incidence, the total field within the upper half-space ($z \leq -h$) of the metagrating can be formulated as [S1]

$$E_{\mathrm{UF}}^{\mathrm{tot}} = E_{\mathrm{in}} e^{-j\beta_0(z+h)} e^{-j\xi_0 y} + \sum_{m=-\infty}^{\infty} E_m^{\mathrm{UF}} e^{-j\xi_m y} e^{j\beta_m(z+h)} \tag{S1}$$

Similarly, the total field within the lower space ($z \geq 0$) of the metagrating is described as

$$E_{\mathrm{LF}}^{\mathrm{tot}} = \sum_{m=-\infty}^{\infty} E_m^{\mathrm{LF}} e^{-j\xi_m y} e^{-j\beta_m z} \tag{S2}$$

In the provided expressions, the abbreviations ‘UF’ and ‘LF’ denote, respectively, the upper and lower space fields generated under forward illumination. E_m^{UF} and E_m^{LF} then represent the amplitudes of the mth order diffraction mode in the upper and lower spaces of the metagrating, respectively. These quantities also correspond to the reflection and transmission diffraction modes, and are defined as

$$E_m^{\mathrm{UF}} = -\frac{\eta_0 k_0}{2\Lambda_y}\left(I_{\mathrm{U}}^{\mathrm{F}}\frac{1+R_m}{\beta_m} + I_{\mathrm{L}}^{\mathrm{F}}\frac{T_m}{\beta_m}e^{j\xi_m d}\right) + \delta_{m0}E_{\mathrm{in}}R_0 \tag{S3}$$

$$E_m^{\mathrm{LF}} = -\frac{\eta_0 k_0}{2\Lambda_y}\left(I_{\mathrm{U}}^{\mathrm{F}}\frac{T_m}{\beta_m} + I_{\mathrm{L}}^{\mathrm{F}}\frac{1+R_m}{\beta_m}e^{j\xi_m d}\right) + \delta_{m0}E_{\mathrm{in}}T_0 \tag{S4}$$

where $I_{\mathrm{U}}^{\mathrm{F}}$ and $I_{\mathrm{L}}^{\mathrm{F}}$ represent the generated line currents on the upper and lower wires in the supercell under forward illumination, respectively.

For backward illumination, the total field in the upper half-space ($z \le -h$) of the metagrating can be can be derived similarly to the forward illuminationas

$$E_{\mathrm{UB}}^{\mathrm{tot}} = \sum_{m=-\infty}^{\infty} E_m^{\mathrm{UB}} e^{-j\xi_m y}\, e^{j\beta_m(z+h)} \tag{S5}$$

The total field in the lower space ($z \ge 0$) of the metagrating is expressed as follows

$$E_{\mathrm{LB}}^{\mathrm{tot}} = E_{\mathrm{in}} e^{-j\beta_0 z} e^{-j\xi_0 y} + \sum_{m=-\infty}^{\infty} E_m^{\mathrm{LB}} e^{-j\xi_m y}\, e^{-j\beta_m z} \tag{S6}$$

In order to differentiate it from the field expressions under forward illumination, the abbreviations ‘UB’ and ‘LB’ are employed to denote the upper and lower space fields generated under backward illumination. Consequently, E_m^{UB} and E_m^{LB} are then formulated as

$$E_m^{\mathrm{UB}} = -\frac{\eta_0 k_0}{2\Lambda_y}\left(I_{\mathrm{U}}^{\mathrm{B}}\frac{1+R_m}{\beta_m} + I_{\mathrm{L}}^{\mathrm{B}}\frac{T_m}{\beta_m}e^{j\xi_m d}\right) + \delta_{m0}E_{\mathrm{in}}T_0 \tag{S7}$$

$$E_m^{\mathrm{LB}} = -\frac{\eta_0 k_0}{2\Lambda_y}\left(I_{\mathrm{U}}^{\mathrm{B}}\frac{T_m}{\beta_m} + I_{\mathrm{L}}^{\mathrm{B}}\frac{1+R_m}{\beta_m}e^{j\xi_m d}\right) + \delta_{m0}E_{\mathrm{in}}R_0 \tag{S8}$$

where $I_{\mathrm{U}}^{\mathrm{B}}$ and $I_{\mathrm{L}}^{\mathrm{B}}$ represent the generated line currents on the upper and lower wires under backward illumination. In the context of backward illumination, where the incidence occurs below the metagratings, the upper diffraction mode E_m^{UB} represents the mth transmission diffraction mode, while E_m^{LB} represents the mth reflection mode.

To ensure that only the 0th diffraction mode propagates while other higher-order modes remain evanescent, the period Λ_y of the metagrating is chosen such that $\Lambda_y < \lambda_0$. The amplitudes of the 0th diffraction modes in the upper and lower regions of the metagrating under both forward and backward illuminations can be represented by $E_0^{\mathrm{UF}} = E_{\mathrm{in}}\mathrm{A}^{\mathrm{UF}}$, $E_0^{\mathrm{LF}} = E_{\mathrm{in}}\mathrm{A}^{\mathrm{LF}}$, $E_0^{\mathrm{UB}} = E_{\mathrm{in}}\mathrm{A}^{\mathrm{UB}}$ and $E_0^{\mathrm{LB}} = E_{\mathrm{in}}\mathrm{A}^{\mathrm{LB}}$, where E_{in} is the incident field amplitude. By selecting different values for A^{UF}, A^{LF}, A^{UB} and A^{LB}, the functionalities of the Janus metagrating can be tailored.

Next, an analysis is conducted on designing the metagratings to achieve different wavefront manipulations under forward illumination. Following the aforementioned discussion, $E_0^{\mathrm{UF}} = E_{\mathrm{in}}\mathrm{A}^{\mathrm{UF}}$ and $E_0^{\mathrm{LF}} = E_{\mathrm{in}}\mathrm{A}^{\mathrm{LF}}$ are substituted into Equations (S3) and (S4). Consequently, the corresponding line currents can be derived as

$$I_{\mathrm{U}}^{\mathrm{F}} = \frac{\Lambda_0}{\eta_0} E_{\mathrm{in}} p' \tag{S9}$$

$$I_{\mathrm{L}}^{\mathrm{F}} = \frac{\Lambda_0}{\eta_0} E_{\mathrm{in}} q' \tag{S10}$$

with

$$p' = 2\frac{(1+R_0)(R_0-\mathrm{A}^{\mathrm{UF}})-T_0(T_0-\mathrm{A}^{\mathrm{LF}})}{(1+R_0)^2-T_0^2} \tag{S11}$$

$$q' = 2\frac{T_0(1+\mathrm{A}^{\mathrm{UF}})-(1+R_0)\mathrm{A}^{\mathrm{LF}}}{(1+R_0)^2-T_0^2} \tag{S12}$$

Then, by combining Equations (S1), (S2), (S9) and (S10), and Ohm's law $E^{\mathrm{tot}} = ZI$, the required load impedance density of the wires on the upper layer $Z_{\mathrm{U}}^{\mathrm{F}}$ and lower layer $Z_{\mathrm{L}}^{\mathrm{F}}$ can be calculated as

$$Z_{\mathrm{U}}^{\mathrm{F}} = \frac{\eta_0}{\Lambda_y}\frac{1+R_0}{p'} - Z_{\mathrm{self}} - \frac{q'}{p'} Z_{\mathrm{mutual}} \tag{S13}$$

$$Z_{\mathrm{L}}^{\mathrm{F}} = \frac{\eta_0}{\Lambda_y}\frac{T_0}{q'} - Z_{\mathrm{self}} - \frac{p'}{q'} Z_{\mathrm{mutual}} \tag{S14}$$

with

$$Z_{\mathrm{self}} = \frac{\eta_0(1+R_0)}{2\Lambda_y} + j\frac{\eta_0}{\lambda_0}\left\{\sum_{m=1}^{\infty}\left[\frac{2\pi(1+R_m)}{j\Lambda_y\beta_m} - \frac{1}{m}\right] - \ln\left(\frac{2\pi r}{\Lambda_y}\right)\right\} \tag{S15}$$

$$Z_{\mathrm{mutual}} = \frac{k_0\eta_0}{2\Lambda_y}\sum_{m=-\infty}^{\infty}\frac{T_m}{\beta_m} e^{-j\xi_m d} \tag{S16}$$

Similarly, the analysis for achieving different wavefront manipulations under backward illumination can also be conducted, following the approach used for forward illumination. Thus, for $E_0^{\mathrm{UB}} = E_{\mathrm{in}}\mathrm{A}^{\mathrm{UB}}$ and $E_0^{\mathrm{LB}} = E_{\mathrm{in}}\mathrm{A}^{\mathrm{LB}}$, the required load impedance density of the wires on the upper layer $Z_{\mathrm{U}}^{\mathrm{B}}$ and lower layer $Z_{\mathrm{L}}^{\mathrm{B}}$ can be derived as

$$Z_{\mathrm{U}}^{\mathrm{B}} = \frac{\eta_0}{\Lambda_y}\frac{T_0}{p''} - Z_{\mathrm{self}} - \frac{q''}{p''} Z_{\mathrm{mutual}} \tag{S17}$$

$$Z_{\mathrm{L}}^{\mathrm{B}} = \frac{\eta_0}{\Lambda_y}\frac{1+R_0}{q''} - Z_{\mathrm{self}} - \frac{p''}{q''} Z_{\mathrm{mutual}} \tag{S18}$$

with

$$p'' = 2\frac{T_0\left(1+\mathrm{A}^{\mathrm{LB}}\right)-(1+R_0)\mathrm{A}^{\mathrm{UB}}}{(1+R_0)^2-T_0^2} \tag{S19}$$

$$q'' = 2\frac{(1+R_0)(R_0-\mathrm{A}^{\mathrm{LB}})-T_0\left(T_0-\mathrm{A}^{\mathrm{UB}}\right)}{(1+R_0)^2-T_0^2} \tag{S20}$$

To actualize the designs of the Janus metagratings, it is imperative to ensure that the design of the wires present consistent load impedances when simultaneously achieving the wavefront manipulations corresponding to forward and backward illumination, *i.e.*, $Z_{\mathrm{U}}^{\mathrm{F}} = Z_{\mathrm{U}}^{\mathrm{B}}$ and $Z_{\mathrm{L}}^{\mathrm{F}} = Z_{\mathrm{L}}^{\mathrm{B}}$. After a series of algebraic simplifications, a simplified equation can be derived as

$$(1 + R_0)(p'' - q') + T_0(q'' - p') = 0 \tag{S21}$$

By solving the Equation (S21), solutions can be obtained that satisfy $Z_{\mathrm{U}}^{\mathrm{F}} = Z_{\mathrm{U}}^{\mathrm{B}}$ and $Z_{\mathrm{L}}^{\mathrm{F}} = Z_{\mathrm{L}}^{\mathrm{B}}$, thereby ensuring the feasibility of the Janus metagrating design scheme for the corresponding function.

When considering absorption under forward illumination and reflection under backward illumination, the parameters are defined as follows: $\mathrm{A}^{\mathrm{UF}} = 0, \mathrm{A}^{\mathrm{LF}} = 0, \mathrm{A}^{\mathrm{UB}} = 0, \mathrm{A}^{\mathrm{LB}} = e^{j\varphi_{\mathrm{LB}}}$. By substituting these values into Equations (S11), (S12), (S19) and (S20), the parameters p', q', p'', q'' can be calculated. Then, by submitting them into Equations (S21) and performing some algebraic simplifications, it is found that Equation (S21) reduces to an identity in this scenario, indicating that the equations of $Z_{\mathrm{U}}^{\mathrm{F}} = Z_{\mathrm{U}}^{\mathrm{B}}$ and $Z_{\mathrm{L}}^{\mathrm{F}} = Z_{\mathrm{L}}^{\mathrm{B}}$ have identical solutions. Therefore, we can opt to solve either one of them. In this case, $Z_{\mathrm{U}}^{\mathrm{F}} = Z_{\mathrm{U}}^{\mathrm{B}}$ is chosen to be solved, and we reach

$$\frac{\eta_0}{\Lambda_y}\left(\frac{1+\Gamma}{p'} - \frac{T}{p''}\right) - \left(\frac{q'}{p'} - \frac{q''}{p''}\right) Z_{\mathrm{mutual}} = 0 \tag{S22}$$

After calculating the desired corresponding parameters of the metagrating using the above equation, these values are then substituted into the set of Equations (S13) and (S14), or (S17) and (S18), to calculate the desired values of load impedance density Z_{U} and Z_{L}.

Text S2. Design of the Janus metagrating with asymmetrically positioned meta-atoms

When the positions of the upper and lower meta-atoms are asymmetric (*i.e.*, $d \neq 0$), Z_{mutual} in Equation (2) of the main text must be replaced by Z_{mutual} given in Equation (S16). In this scenario, the two equations $\Re(J) = 0$ and $\Im(J) = 0$ with respect to the complex number $J = \frac{\eta_0}{\Lambda_y}\left(\frac{1+R_0}{p'} - \frac{T_0}{p''}\right) - \left(\frac{q'}{p'} - \frac{q''}{p''}\right) Z_{\mathrm{mutual}}$ contain four unknowns: d, Λ_y, h and φ_{LB}. For this case, we can choose two parameters arbitrarily. Considering the sparsity and low profile of the Janus metagrating, we determine Λ_y and h from these four parameters.

Similarly, when selecting these parameters, we need to avoid situations where the real part of the load impedance obtained from the final computation is negative. After a judicious parameter screening, the period of the metagrating Λ_y and the thickness of the substrate h are chosen as Λ_y = 16 mm (greater than $\lambda_0/2$) and h = 2 mm (lower than $\lambda_0/10$), respectively. Subsequently, the horizontal spacing between the two wires d = 1.46 mm and the parameter φ_{LB} = 2.1951 rad can be calculated according to $\Re(J) = 0$ and $\Im(J) = 0$. The required impedance densities of the two wires are then calculated as Z_{U} = - j4.3926 η/λ and Z_{L} = (0.2752 – j3.8552) η/λ, respectively.

To realize these values of the load impedance, we use meta-atoms composed of microstrip line capacitor and chip resistor as in the main text to construct the supercell shown in **Figure S2**. Finally, the corresponding parameters of the meta-atoms are calculated as: L_{U} = 3.78 mm, L_{L} = 4.31 mm, R_{L} = 10.37 Ω, which are further optimized in simulations, and the final optimized parameters are L_{U} = 3.59 mm, L_{L} = 4.01 mm, R_{L} = 7.57 Ω,. All values of the Janus metagrating supercell are listed in **Table S1**. Compared to the symmetric position of the meta-atoms in the main text, the meta-atoms here are not symmetric, introducing an additional design degree of freedom, which is the parameter d. This enables more flexibility in choosing the substrate thickness; for instance, the substrate thickness here (2 mm) is thinner than that in the main text (2.5 mm).

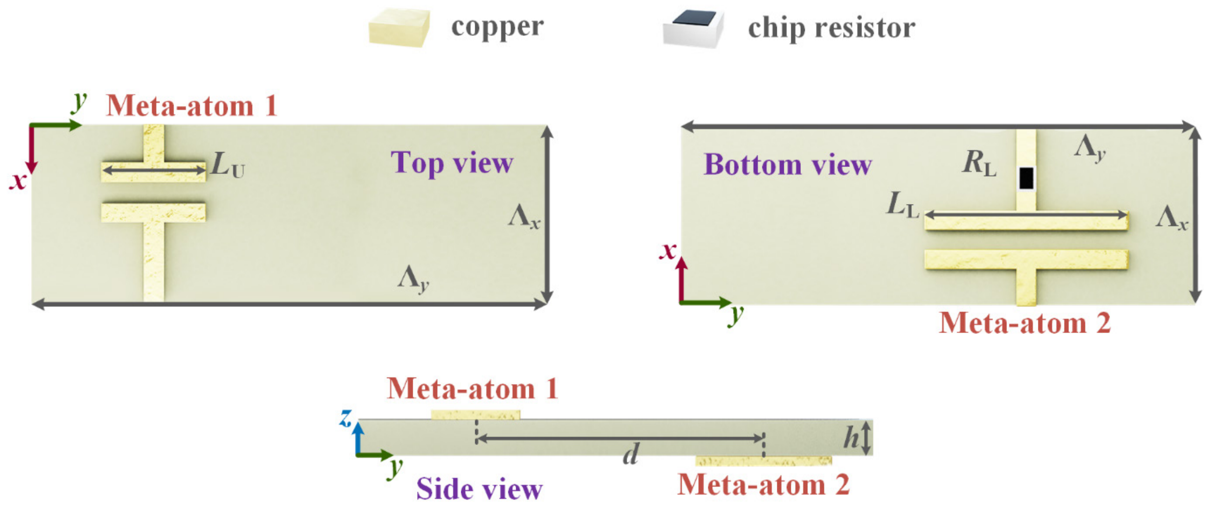

Figure S2. Individual views of the Janus metagrating supercell composed of realistic meta-atoms with asymmetrically positioned upper and lower meta-atoms.

Table S1. Parameters of Janus metagrating supercell with asymmetrically positioned meta-atoms

Parameters	Calculated	Optimized
h (mm)	2	2
L_U (mm)	3.78	3.59
L_L (mm)	4.31	4.01
R_L (Ω)	10.37	7.57

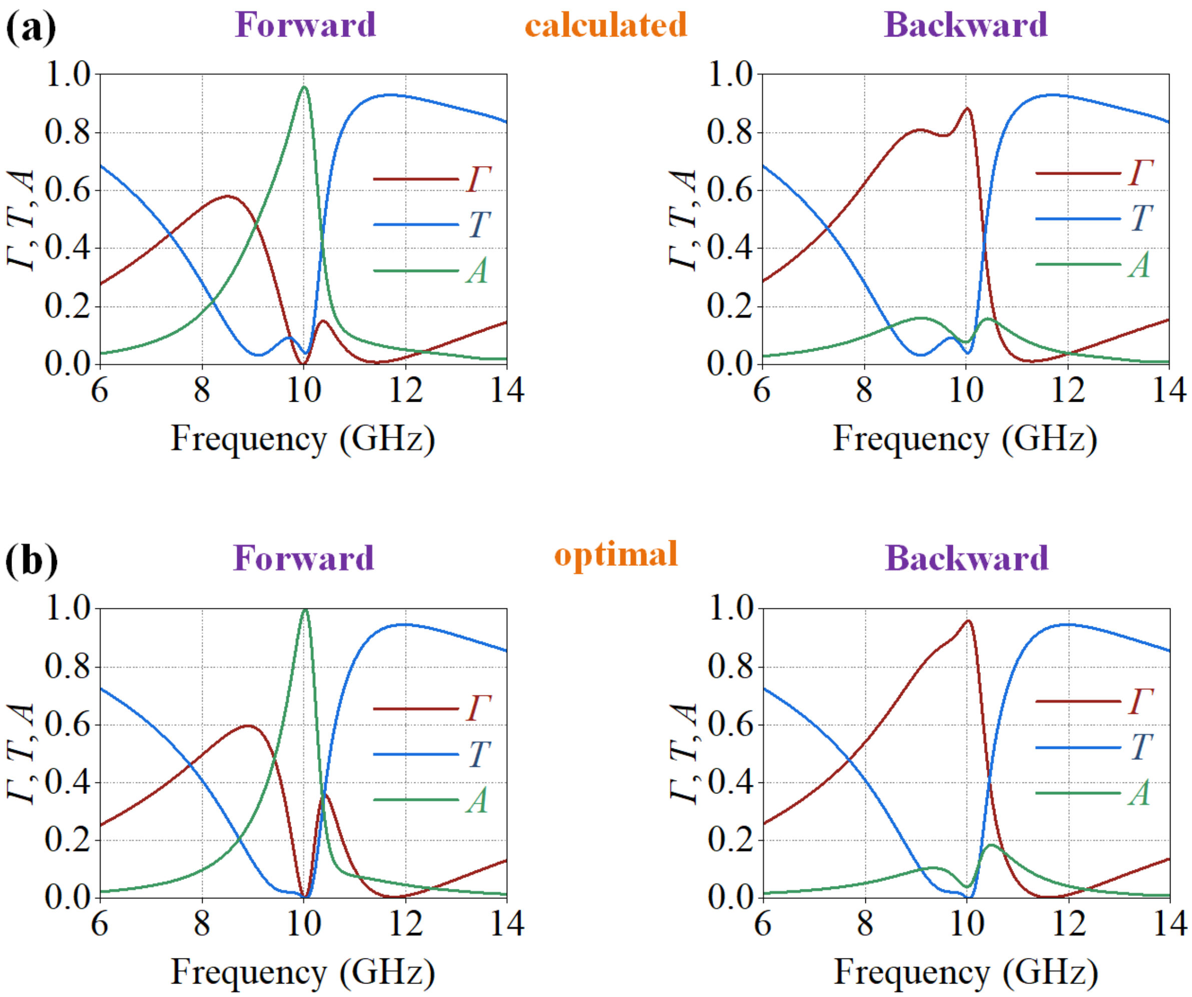

Figure S3. Simulation results of the Janus metagrating supercell with asymmetrically positioned meta-atoms for asymmetric absorption and reflection a) Calculated Parameters. b) Optimized Parameters.

The simulation results for both forward and backward illuminations of the Janus metagrating supercell with calculated parameters and optimal parameters are presented in **Figure S3**. For the supercell with calculated parameters, the maximum absorption under forward illumination is 95.70% at 10.01 GHz, and the maximum reflection under backward illumination is 88.25% at 10.02 GHz. After optimization, the supercell achieves 100% absorption under forward illumination at 10 GHz and 96% reflection efficiency under backward illumination at 10 GHz. When comparing the simulation results of the supercell obtained with unoptimized parameters

shown in Figure 3 of the main text and in Figure S3a, no deviation in operating frequency is observed from the initial target with the configuration of asymmetrically positioned meta-atoms. This indicates that when the positions of the two meta-atoms are not aligned vertically, their coupling is considerably reduced and the accuracy of the analytical calculations is improved. Moreover, compared to the final optimal simulation results shown in Figure 4c of the main text, the reflection loss shown in Figure S3b is reduced from 5% to 4%, attributed to the use of a smaller resistor value R_L, as shown in Table S1. Consequently, the smaller resistance value results in a narrower absorption bandwidth under forward illumination.

Text S3. Performance evaluation of Janus metagratings: absorption bandwidth-to-thickness ratio

To further evaluate the performance of Janus metagratings in terms of the absorption bandwidth-to-thickness ratio, we compare it with the theoretical limit for transparent layers, as derived in Ref. [S1]. According to [S1], the upper bound for the bandwidth-to-thickness ratio of a transparent layer is given by

$$\frac{\Delta\lambda}{h} \leq \frac{\pi^2(\varepsilon_r+\mu_r-2)}{|ln(1-A)|} \tag{S23}$$

where $\Delta\lambda$ is the bandwidth corresponding to the absorption rate A, while ε_r and μ_r denote the relative permittivity and permeability of the substrate, respectively.

Then, the normalized absorption bandwidth-to-thickness ratio is then defined as

$$\eta = \frac{\Delta\lambda/h}{\pi^2(\varepsilon_r+\mu_r-2)/|ln(1-A)|} \tag{S24}$$

In our analysis, we select A = 90%, such that $\Delta\lambda$ represents the wavelength range where the absorption rate exceeds 90%. Based on this definition, the normalized absorption bandwidth-to-thickness ratio of the Janus metagrating is calculated as 0.054 (simulation) and 0.098 (experiment), demonstrating its performance relative to the theoretical limit.

[S1] W. J. Padilla, Y. Deng, O. Khatib, and V. Tarokh, "Fundamental absorption bandwidth to thickness limit for transparent homogeneous layers," *Nanophotonics*, vol. 13, no. 9, pp. 1623-1629, 2024.
